# The complete mitochondrial genome of Mong Cai pig (*Sus scrofa*) in Vietnam

**DOI:** 10.1080/23802359.2016.1155424

**Published:** 2016-03-28

**Authors:** Thuy Nhien Thi Tran, Pan Ni, Jianhai Chen, Thuy Thi Le, Kemp Steve, Jianlin Han, Haiyan Wang, Shuhong Zhao

**Affiliations:** a Key Lab of Agricultural Animal Genetics and Breeding, Ministry of Education, College of Animal Science and Veterinary Medicine, Huazhong Agricultural University, Wuhan, PR China;; b National Institute of Animal Sciences, Hanoi, Vietnam;; c International Livestock Research Institute (ILRI), Nairobi, Kenya;; d CAAS-ILRI Joint Laboratory on Livestock and Forage Genetic Resources, Institute of Animal Science, Chinese Academy of Agricultural Sciences (CAAS), Beijing, China;; e The Cooperative Innovation Center for Sustainable Pig Production, Huazhong Agricultural University, Wuhan, PR China

**Keywords:** Mong Cai pig, mitochondrial genome, phylogeny

## Abstract

The Mong Cai pig is an indigenous breed and popularly raised as maternal line in northern Vietnam. In this study, the complete mitochondrial genome sequence of the Mong Cai pig is reported. The total length of this mitochondrial genome is 16 632 bp, including 1 non-coding control region (D-loop region), two ribosomal RNA genes, 13 protein-coding genes and 22 transfer RNA genes. The phylogenetic tree of 162 pig complete mitogenomes reveals a very close relationship between Mong Cai pig in Vietnam and Bama miniature pig in southern China. This complete mitochondrial genome sequence of Mong Cai pig is useful to further genetic studies on adaptation and performance.

Mong Cai pig is one of the unique and indigenous pig breeds in Vietnam. It is originated from Mong Cai district of Quang Ninh province and is farmed widely in northern Vietnam. With its high fertility, Mong Cai pigs are commonly used as basic sows for crossing with Yorkshire and Landrace pig breeds (Tra 2003). Mong Cai pigs are very important to the local economy in northern Vietnam due to their better reproductive performance than international breeds like Large White under small farm conditions based on inexpensive plant diets. However, the complete mitochondrial DNA sequences for Mong Cai pigs are scanty.

Here, we sequenced the complete mitochondrial genome of a Mong Cai pig in Son La province, Vietnam (coordinates: 21°12'29.9″N, 103°05'24.8″E; GenBank accession no. KU556691). The ear sample was stored in the Key Lab of Agricultural Animal Genetics and Breeding, College of Animal Science and Veterinary Medicine, Huazhong Agricultural University (ID: 20151011mongcai14). The sequence was amplified using 35 pairs of primers (Li et al. 2016). MEGA6 program (Tamura et al. 2013) was used to build the phylogenetic tree of 162 complete pig mitochondrial DNA sequences, including 161 retrieved from the GenBank. Considering the sequences to be closely related, we used the UPGMA algorithm, a distance method that is very suitable for population data (Yang 2014), to infer their phylogenetic relationship (Figure 1). The Bayes tree generated by Mrbayes 3 program (Ronquist & Huelsenbeck 2003) with mixed model was used to double-check the resulting tree topology.

**Figure 1.**
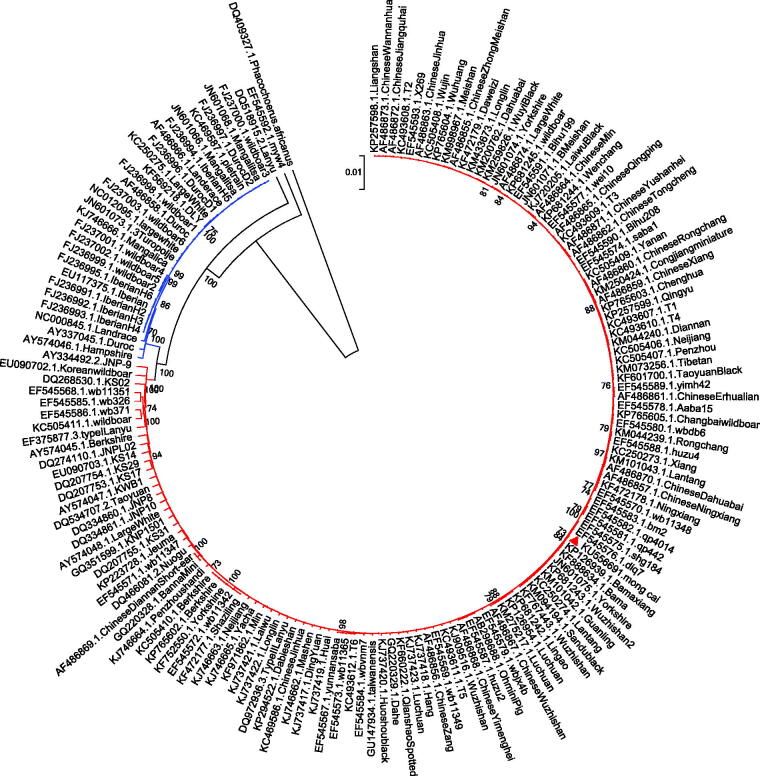
The UPGMA phylogenetic tree of 162 pig complete mitochondrial DNA sequences. The African warthog (*Phacochoerus africanus*) is included as an outgroup. The bootstrap supports above 70% are shown. The two clades represent European and Asian wild boars, local and commercial pig breeds, respectively (European clade is from FJ237000.1.Wildboar3 to AY574046.1.Hampshire, and Asia clade is from AY334492.2.JNP-9 to KP257598.1.Liangshan). The Mong Cai pig is indicated with a triangle.

The total length of Mong Cai pig mitochondrial genome is 16 632 bp with the base composition of 34.61% for A, 25.82% for T, 26.19% for C and 13.37% for G in the order A > C > T > G. It has a feature rich in A + T (60.43%) similar to other mammalian mitochondrial genomes (Lin et al. 1999). This mitochondrial DNA sequence is composed of a typical structure, including 1 non-coding control region (D-loop region), 2 ribosomal RNA genes, 13 protein-coding genes and 22 transfer RNA genes. The phylogenetic trees generated by the UPGMA distance and the Bayes method exhibited a similar topology which revealed two major clusters, representing wild boars, local and commercial pig breeds from Europe and Asia. All the indigenous pigs were clustered in line with their geographical locations, whereas some commercial breeds such as Yorkshire, Berkshire and Large White were mixed into the Asian clade, indicating an Asian contribution to the maternal lines of European commercial breeds. The Mong Cai pig was clustered into the Asian clade. Interestingly, it showed a very close relationship to Bama miniature pig in Guangxi province, southern China. This specific phylogenetic position can be explained by their close spatial distance and extensive human interaction between northern Vietnam and Guangxi province, China.
